# The threshold effect of fasting blood glucose levels on the risk of delivering macrosomia in gestational diabetes mellitus patients

**DOI:** 10.3389/fendo.2025.1587306

**Published:** 2025-05-29

**Authors:** Yuan Li, Yumin Zhu, Wenjun Zheng, Yumei Gao, Ruili Huang, Tingting Yang, Ying Chen

**Affiliations:** Department of Gynaecology and Obstetrics, The First People’s Hospital of Shangqiu, Clinical College of Xuzhou Medical University, Shang Qiu, Henan, China

**Keywords:** gestational diabetes mellitus, fasting blood glucose, macrosomia, threshold effect, pregnancy

## Abstract

**Background:**

Gestational diabetes mellitus (GDM) is a prevalent condition during pregnancy, and macrosomia is a recognized risk associated with it. However, the specific relationship between fasting blood glucose (FBG) levels and the risk of macrosomia in GDM, particularly any potential thresholds for this relationship, remains unclear.

**Methods:**

This retrospective cohort study analyzed data from 7,957 pregnant women who underwent antenatal care and delivered at The First People’s Hospital of Shangqiu between February 1, 2018, and December 30, 2022. Participants were stratified into three groups based on FBG levels: <5.1 mmol/L, 5.1–7 mmol/L, and ≥7 mmol/L. Multivariable logistic regression analyses were performed to assess the association between FBG levels and the risk of macrosomia. Two-piecewise regression models were applied to identify a threshold for the FBG-macrosomia relationship.

**Results:**

The prevalence of macrosomia increased substantially with increasing FBG levels (P < 0.001). The adjusted multivariable logistic regression analyses revealed that compared to women with FBG levels <5.1 mmol/L, those with FBG levels of 5.1–7 mmol/L and ≥7 mmol/L had 4.69 (95% CI: 4.06-5.42) and 8.65 (95% CI: 7.31-10.23) times higher risk of macrosomia, respectively (both P < 0.001). Two-piecewise regression models identified a threshold of 8.037 mmol/L. Below this threshold, each unit increase in FBG was associated with a 1.93-fold increase in the odds of macrosomia (95% CI: 1.83-2.04, P < 0.001). Above this threshold, the association was no longer statistically significant (OR = 1.04, 95% CI: 0.90-1.21, P = 0.587). Furthermore, the stratified analysis also showed a positive association between FBG level and macrosomia.

**Conclusion:**

There is a nonlinear relationship between FBG levels during pregnancy and the risk of macrosomia in GDM women, with a potential threshold effect at 8.037 mmol/L. Below the threshold, macrosomia prevalence markedly rises with elevated FBG levels, whereas above it, the association loses significance, implying a potential saturation at very high glucose levels.

## Introduction

Gestational diabetes mellitus (GDM) is a significant metabolic disorder occurring during pregnancy, posing substantial risks to both the mother and the developing fetus (1, 2). One of the most concerning complications associated with GDM is macrosomia, or excessive fetal growth, which can lead to various adverse outcomes such as difficult delivery, shoulder dystocia, and neonatal hypoglycemia (3, 4). Controlling fasting blood glucose (FBG) levels during pregnancy is crucial in managing GDM and preventing macrosomia.

While previous investigations have established a general correlation between elevated FBG levels and macrosomia risk (5, 6), critical knowledge gaps persist. A landmark study by Landon et al. demonstrated linear associations between maternal glycemia and fetal overgrowth, yet their diagnostic frameworks primarily relied on single-timepoint measurements rather than longitudinal glycemic patterns (7). Subsequent studies by Zhang et al. and Tong et al. attempted to establish a correlation between FBG and birth weight, but their findings showed marked geographical variability, potentially constrained by heterogeneous diagnostic criteria and inconsistent measurement protocols (8, 9). Notably, none of these studies systematically evaluated the specific FBG threshold for GDM patients with the risk of macrosomia, especially when FBG is taken from the GDM average measured three times after treatment.

It is well-established that managing gestational diabetes emphasizes stringent glycemic targets during pregnancy, yet not all patients are able to maintain their FBG levels within the optimal range (10, 11). McIntyre et al. found that there is a lack of uniform worldwide consensus on the hyperglycemic threshold levels that warrant a diagnosis of GDM and subsequent treatment during pregnancy, optimal management of both mother and infant during long-term follow-up remains challenging (12). Persistent discrepancies exist between major international guidelines, particularly between the National Institute for Health and Care Excellence and the World Health Organization diagnostic protocols, this inconsistency stems from fundamental methodological variations across studies. There is marked population heterogeneity in existing studies, most thresholds are derived from European cohorts and lack validation in Asian populations. Moreover, there’s inconsistency in the diagnostic gold standard. Guidelines vary in their emphasis on postprandial versus fasting glucose levels (13, 14). These challenges underscore the urgent need to establish clinically relevant FBG thresholds through robust epidemiological evidence. In this investigation, we conducted longitudinal monitoring of FBG levels from GDM diagnosis through delivery, employing triplicate measurements to enhance glycemic status assessment reliability. Our analysis focuses on elucidating the quantitative relationship between third-trimester FBG control and macrosomia risk, aiming to establish evidence-based glycemic targets for optimizing neonatal outcomes in this high-risk population.

## Materials and methods

### Study design and population

This retrospective cohort study included 7,957 pregnant women who received antenatal care and delivered at The First People’s Hospital of Shangqiu between February 1, 2018, and December 30, 2022. Pregnant women diagnosed with GDM during their antenatal care visits were eligible for inclusion. Data were collected from electronic medical records and antenatal care charts. The following variables were recorded for each participant: age, education level, number of pregnancies, pre-pregnancy body mass index (BMI), smoking status, FBG levels during pregnancy, and the occurrence of macrosomia. Macrosomia was defined as a birth weight exceeding 4000 grams. The study protocol was consistent with ethical principles and received approval from the Ethics Committee of The First People’s Hospital of Shangqiu (No: SYQ1033992). Given the retrospective nature of the study, the Ethical Committee of the First People’s Hospital of Shangqiu waived the requirement of informed consent. The present study is in compliance with the Declaration of Helsinki.

**Figure 1:**
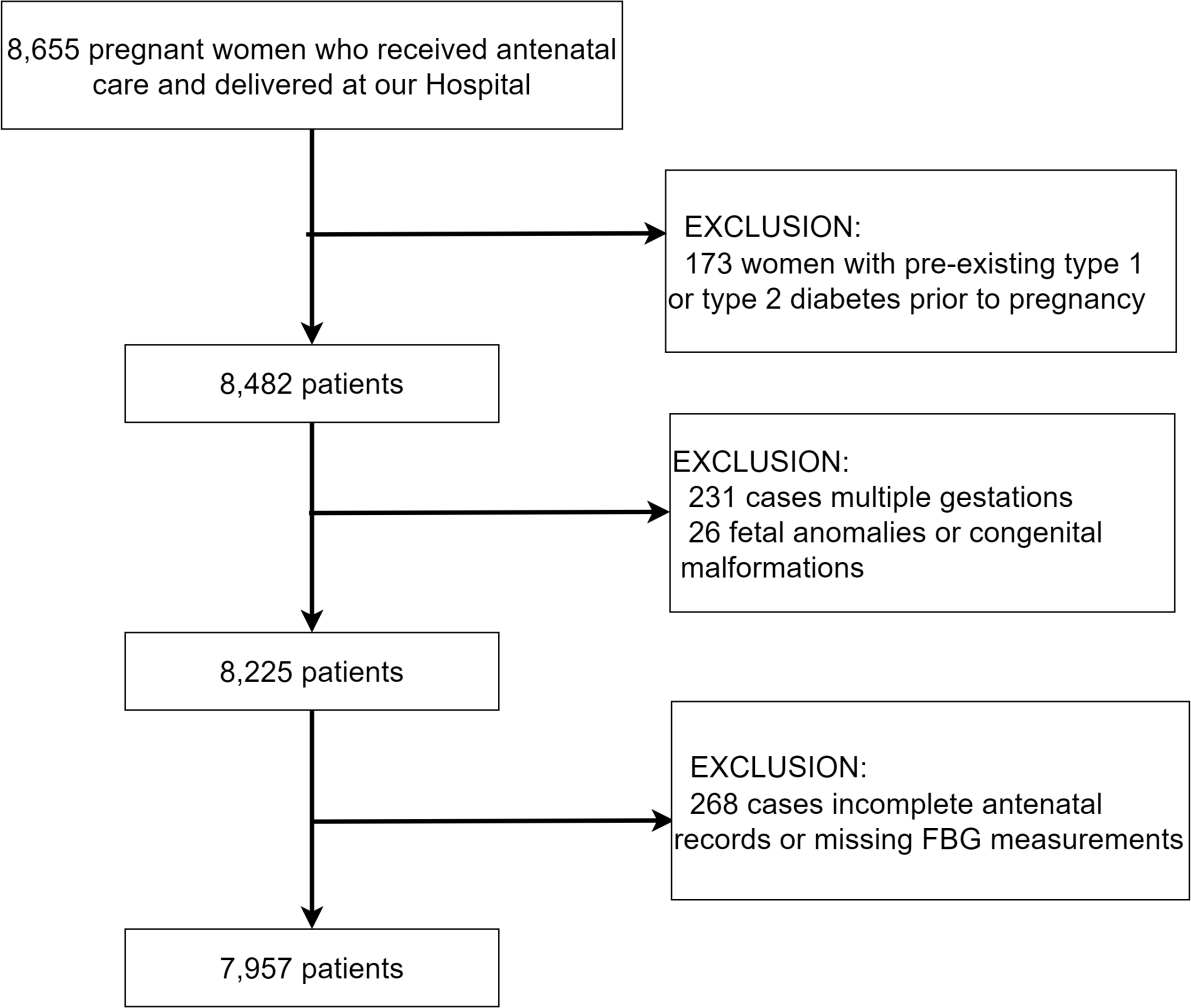
The technology roadmap.

Inclusion Criteria: Pregnant women with a confirmed diagnosis of GDM based on the International Association of Diabetes and Pregnancy Study Groups (IADPSG) criteria during the second trimester of pregnancy; complete antenatal records including at least three FBG measurements from the time of GDM diagnosis until delivery; singleton pregnancies with a known birth outcome. Exclusion Criteria: Women with pre-existing type 1 or type 2 diabetes prior to pregnancy; multiple gestations; incomplete antenatal records or missing FBG measurements; fetal anomalies or congenital malformations. The technical roadmap is detailed in **Figure 1**.

### GDM diagnosis

GDM was diagnosed according to the IADPSG criteria, which define GDM as one or more of the following: fasting plasma glucose ≥ 5.1 mmol/L, 1-hour plasma glucose ≥ 10.0 mmol/L, or 2-hour plasma glucose ≥ 8.5 mmol/L during a 75-gram oral glucose tolerance test performed between 24 and 28 weeks of gestation (15).

### FBG measurement

For each woman included in the study, three FBG measurements were obtained during the period from GDM diagnosis to delivery. The average of these three measurements was calculated and used to stratify women into three groups: FBG < 5.1 mmol/L, 5.1–7 mmol/L, and ≥ 7 mmol/L.

### Statistical analysis

Statistical analysis was performed with SPSS version 26.0 (IBM, Chicago, USA) and R (version 4.3.2). Descriptive statistics were used to summarize the baseline characteristics of the study population. Categorical data were reported as percentages, The chi-square test was applied to assess statistical differences for categorical variables. Multivariable logistic regression analyses were performed to assess the association between FBG levels and the risk of macrosomia, with adjustments for potential confounders including age, BMI, education level, number of pregnancies, and smoking status. Following the Strengthening the Reporting of Observational Studies in Epidemiology (STROBE) recommendations, three models were tested: a crude model without other covariates, Model 1 adjusted for age and BMI, and Model 2 adjusted for age, BMI, education, number of pregnancies, and smoking status.

To identify potential thresholds for the FBG-macrosomia relationship, two-piecewise regression models were applied. These models evaluated the relationship between FBG levels and macrosomia risk below and above a threshold value, which was determined based on statistical significance and goodness of fit. Subgroup analyses were conducted to explore the association between FBG levels and the risk of macrosomia within specific demographic and clinical strata, including age, education, BMI, and smoking status. P-value < 0.05 was considered statistically significant.

## Results

This study analyzed data from 7,957 pregnant women who underwent antenatal care and delivered at The First People’s Hospital of Shangqiu between February 1, 2018, and December 30, 2022. The study population was stratified into three groups based on FBG levels: <5.1 mmol/L (n=3614), 5.1–7 mmol/L (n=3120), and ≥7 mmol/L (n=1223). There were significant differences in education level (p=0.032) and BMI (p<0.001) across the three FBG groups, but age, number of pregnancies, and smoking status did not significantly differ. A substantial increase in the prevalence of macrosomia was observed with increasing FBG levels. Specifically, the prevalence of macrosomia was 7.9% in the <5.1 mmol/L group, 28.2% in the 5.1–7 mmol/L group, and 42.1% in the ≥7 mmol/L group, resulting in a highly significant difference (P < 0.001) (**Table 1**).

**Table 1:** Comparison of three groups of different FBG in patients with clinical data.

Variables	Total (n = 7957)	<5.1 mmol/L (n = 3614)	5.1~7 mmol/L (n = 3120)	≥7 mmol (n = 1223)	*P* value
Age, n (%)					0.080
< 29 year	2453 (30.8)	1097 (30.4)	969 (31.1)	387 (31.6)	
30~35 year	2213 (27.8)	1058 (29.3)	834 (26.7)	321 (26.2)	
36~40 year	1575 (19.8)	686 (19)	622 (19.9)	267 (21.8)	
>40 year	1716 (21.6)	773 (21.4)	695 (22.3)	248 (20.3)	
Education, n (%)					0.032
Hish school and lower	4486 (56.4)	2097 (58)	1729 (55.4)	660 (54)	
College graduate	3152 (39.6)	1365 (37.8)	1269 (40.7)	518 (42.4)	
Post-graduate	319 (4.0)	152 (4.2)	122 (3.9)	45 (3.7)	
Number pregnancy, n (%)					0.275
≤1	2772 (34.8)	1290 (35.7)	1074 (34.4)	408 (33.4)	
>2	5185 (65.2)	2324 (64.3)	2046 (65.6)	815 (66.6)	
BMI, n (%)					< 0.001
<23.9 kg/m^2^	2370 (29.8)	1150 (31.8)	901 (28.9)	319 (26.1)	
23.9-29.9 kg/m^2^	2252 (28.3)	975 (27)	920 (29.5)	357 (29.2)	
29.9–35 kg/m^2^	1872 (23.5)	877 (24.3)	717 (23)	278 (22.7)	
>35 kg/m^2^	1463 (18.4)	612 (16.9)	582 (18.7)	269 (22)	
Smoking, n (%)					0.132
No	7307 (91.8)	3322 (91.9)	2879 (92.3)	1106 (90.4)	
Yes	650 (8.2)	292 (8.1)	241 (7.7)	117 (9.6)	
Macrosomia, n (%)					< 0.001
No	6275 (78.9)	3327 (92.1)	2240 (71.8)	708 (57.9)	
Yes	1682 (21.1)	287 (7.9)	880 (28.2)	515 (42.1)	

BMI, body mass index; Data presented as unweighted numbers (weighted percentage) for categorical variables.

Multivariable logistic regression analyses consistently demonstrated a positive relationship between higher FBG levels and the risk of macrosomia. In the fully adjusted model (Model 2), compared to women with FBG levels <5.1 mmol/L, those with FBG levels of 5.1–7 mmol/L and ≥7 mmol/L had 4.69 (95% CI: 4.06-5.42) and 8.65 (95% CI: 7.31-10.23) times higher risk of macrosomia, respectively (both P < 0.001). A trend test confirmed a linear increase in the risk of macrosomia with rising FBG levels (P < 0.001) (**Table 2**).

**Table 2:** Multivariable logistic regression analyses of FBG and macrosomia.

Variables	Total (n)	N event%	Crude OR (95% CI)	*P* value	Model 1 OR (95% CI)	*P* value	Model 2 OR (95% CI)	*P* value
FBG	7957	1682 (21.1)	1.59 (1.53~1.64)	<0.001	1.59 (1.53~1.65)	<0.001	1.60 (1.54~1.66)	<0.001
Subgroup (tertiles)								
<5.1 mmol/L	3614	287 (7.9)	1(Ref)		1(Ref)		1(Ref)	
5.1~7 mmol/L	3120	880 (28.2)	4.55 (3.94~5.26)	<0.001	4.57 (3.96~5.28)	<0.001	4.69 (4.06~5.42)	<0.001
≥7 mmol	1223	515 (42.1)	8.43 (7.15~9.95)	<0.001	8.49 (7.19~10.03)	<0.001	8.65 (7.31~10.23)	<0.001
Trend test			2.89 (2.67~3.13)	<0.001	2.90 (2.68~3.14)	<0.001	2.93 (2.71~3.17)	<0.001

BMI, body mass index.

Crude model: no other covariates were adjusted.

Model 1: adjusted for Age, BMI.

Model 2: adjusted for Age, BMI, Education, Number pregnancy, Smoking.

**Figure 2:**
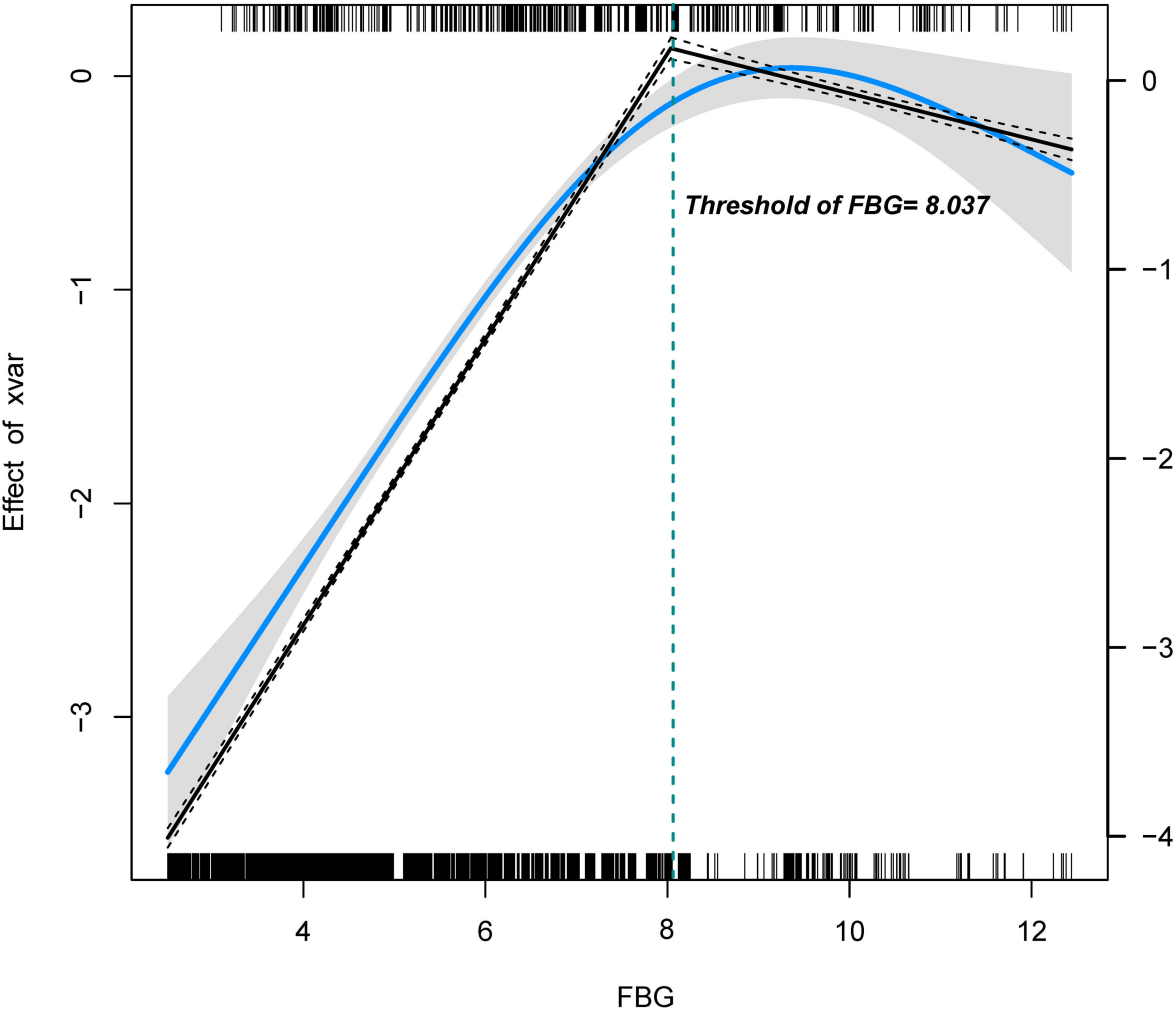
Association between FBG and macrosomia using two-piecewise regression. The solid blue line indicates the predicted value and the grey areas indicate the 95% CI. The restricted cubic spline model was adjusted for Age, BMI, Education, Number of pregnancies, and Smoking.

To identify a potential threshold for the FBG-macrosomia relationship, two-piecewise regression models were applied (**Figure 2**). The analysis identified a significant threshold at 8.037 mmol/L, beyond which the risk of macrosomia did not further increase significantly. Below this threshold, each unit increase in FBG was associated with a 1.93-fold increase in the odds of macrosomia (95% CI: 1.83-2.04, P < 0.001). Above this threshold, the association was no longer statistically significant (OR = 1.04, 95% CI: 0.90-1.21, P = 0.587), suggesting a potential plateau or saturation effect (**Table 3**).

**Table 3:** Association between FBG and macrosomia using two-piecewise regression models.

Threshold of FBG	OR	95%CI	*P* value
≤8.037 mmol/L	1.931	1.832~2.036	<0.001
>8.037 mmol/L	1.042	0.899~1.207	0.587
Non-linear test			<0.001

Adjusted for Age, BMI, Education, Number pregnancy, Smoking.

**Figure 3:**
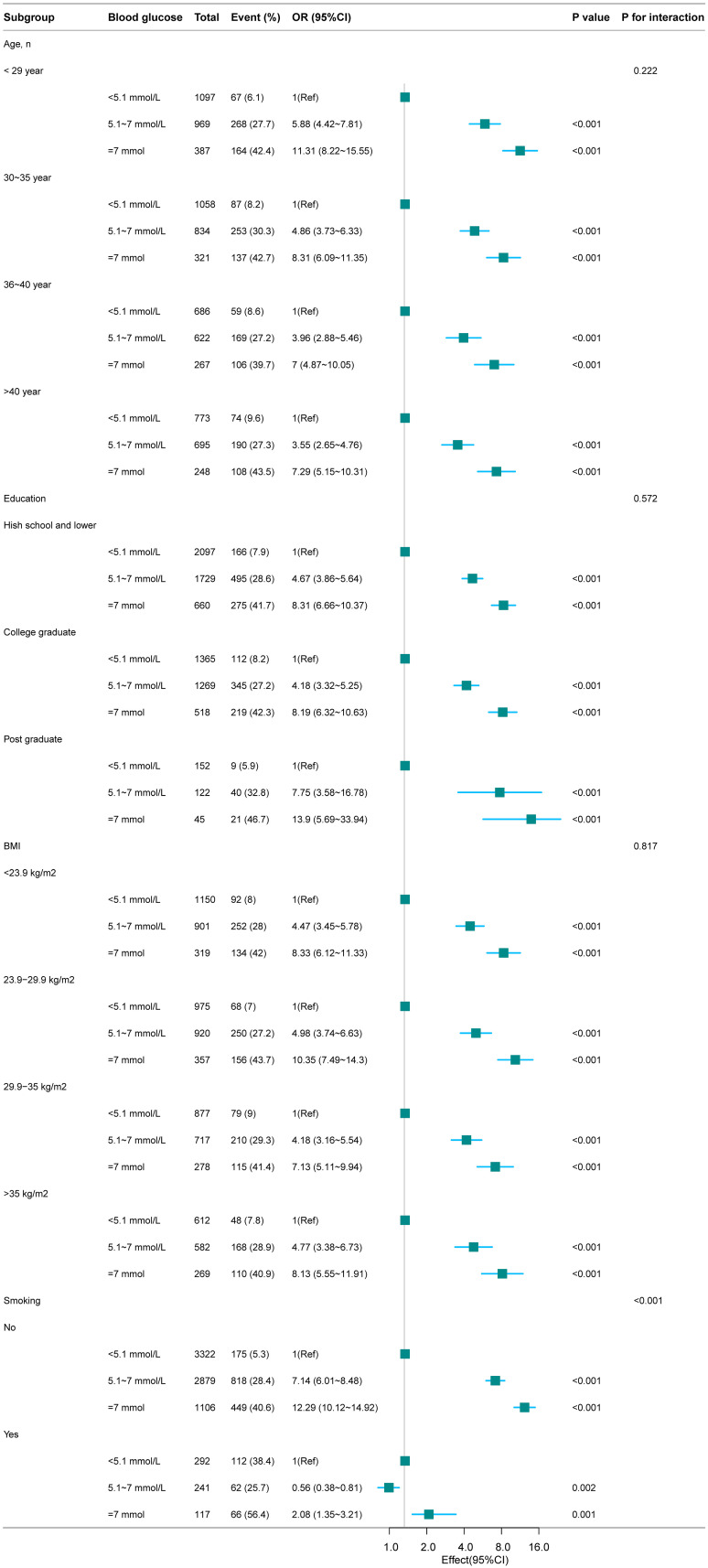
Association between FBG and macrosomia according to the subgroup analyses. Except for the stratification factor itself, the stratifications were adjusted for all variables (Age, BMI, Education, Smoking).

Stratified analyses by age, education, BMI, and smoking status revealed that the risk of macrosomia increased significantly with higher FBG levels across all subgroups. Notably, smokers had a markedly higher prevalence of macrosomia even within the lowest FBG group (<5.1 mmol/L), highlighting the importance of considering smoking status in clinical management (**Figure 3**).

## Discussion

The incidence of GDM is notably high, and its implications are profound (16). Studies have shown that inadequate control of FBG levels is associated with an increased risk of macrosomia (17, 18), a condition characterized by excessive fetal growth. This complication not only poses significant challenges to the maternal delivery process, including difficult labor and shoulder dystocia, but also threatens the fetus, potentially leading to neonatal hypoglycemia and other adverse outcomes (19, 20). Therefore, it is imperative to prioritize effective FBG control in GDM management to mitigate these risks and optimize maternal and fetal health outcomes. Our study revealed a nonlinear relationship between fasting FBG levels during pregnancy and the risk of macrosomia in women with gestational diabetes mellitus, with a potential threshold effect observed at 8.037 mmol/L. Specifically, below this threshold, each unit increase in FBG was associated with a 1.93-fold increase in the odds of macrosomia. Conversely, above this threshold, the association between FBG and macrosomia became statistically insignificant, suggesting a potential saturation effect or the involvement of other mitigating factors at extremely high glucose levels.

Our findings emphasize a substantial rise in the prevalence of macrosomia with increasing FBG levels (P < 0.001), aligning with prior research that established a connection between hyperglycemia and fetal overgrowth, corroborating the observations made by Zhang et al (8). Additionally, the two-piecewise regression models identified a threshold of 8.037 mmol/L, this novel discovery contributes to the existing literature by indicating that FBG levels reaching a specific threshold may not further exacerbate the risk of macrosomia. The exact mechanism by which hyperglycemia leads to macrosomia in GDM women remains elusive. Nevertheless, several studies have postulated that hyperglycemia may induce fetal hyperinsulinemia, thereby stimulating fetal growth and adiposity (21, 22). Furthermore, hyperglycemia may facilitate increased nutrient transport to the fetus, further promoting fetal growth (23). Our study supports these mechanisms by demonstrating a dose-response relationship between FBG levels and the risk of macrosomia. However, the absence of a significant association above the identified threshold hints at the possibility of additional mechanisms or compensatory responses operative at very high glucose levels, necessitating further investigation.

Consensus on optimal glycemic control targets for GDM pregnant women remains unsettled. In 2013, the American Association of Clinical Endocrinologists’ clinical practice guidelines suggested controlling fasting plasma glucose to <5.0 mmol/L while avoiding hypoglycemia (24). In 2015, the National Institute for Health and Care Excellence (NICE) guidelines in the UK recommended controlling 2-hour postprandial glucose to <6.4 mmol/L (25). Regarding the definition of hyperglycemia, the IADPSG utilized data from the HAPO study to establish a diagnostic threshold for GDM at FBG levels that elevate the risk of adverse pregnancy outcomes by 75% compared to average levels, with a fasting plasma glucose cutoff of 5.1 mmol/L (26). Studies examining FBG fluctuations in normal pregnant women have revealed that their peak postprandial glucose levels during weeks 28 to 38 of pregnancy remain below 5.8 mmol/L (27, 28). For GDM pregnant women, the ADA and FIGO currently recommend glycemic control targets of fasting, pre-meal, or bedtime glucose <5.3 mmol/L, 1-hour postprandial <7.8 mmol/L, or 2-hour postprandial <6.7 mmol/L (15, 29). These discrepancies are likely attributable to heterogeneous clinical objectives, divergent patient demographics, and methodological inconsistencies across validation studies. Specifically, the NICE guidelines prioritize diagnostic sensitivity for early detection in predominantly UK/European cohorts, whereas the ADA recommendations emphasize specificity optimization to minimize false-positive diagnoses in advanced-stage US populations. Despite these nuanced differences, both sets of guidelines consistently advocate for stringent FBG control in pregnant women with GDM, as elevated FBG levels during pregnancy are strongly associated with adverse perinatal outcomes.

Our findings align with the glycemic control targets recommended by the ADA and FIGO guidelines, demonstrating a significant association between maternal FBG levels during pregnancy and neonatal outcomes in GDM patients. Notably, the present study revealed a positive correlation between FBG levels below the identified threshold of 8.037 mmol/L and macrosomia incidence. However, this association lost statistical significance when FBG exceeded this critical value, though this observation should not be misinterpreted as justifying glycemic negligence. Substantial evidence confirms that suboptimal glycemic control in gestational diabetes predisposes to adverse perinatal outcomes (30–32). Elevated glucose levels in pregnant women traverse the placenta, stimulating fetal pancreatic β-cell proliferation and insulin secretion, which can adversely impact fetal health (33, 34). Moreover, high glucose levels heighten the risk of reproductive tract infections, intrauterine infections, and subsequent inflammatory cytokine production, potentially leading to preterm birth (35). This preterm birth phenomenon associated with excessive FBG levels might partially explain the attenuated correlation between supra-threshold hyperglycemia and macrosomia. Hyperglycemia is frequently accompanied by insulin resistance, and elevated insulin levels stimulate sympathetic nerve excitation, simultaneously, high FBG levels damage endothelial cells, leading to vasoconstriction and elevated blood pressure (12, 36). Pregnant women with high FBG levels are thus at an increased risk of adverse perinatal outcomes, including macrosomia, preterm birth, and preeclampsia.

One of the strengths of our study lies in its large sample size, which enabled us to stratify participants into three groups based on FBG levels and conduct subgroup analyses stratified by age, education, BMI, and smoking status to assess the association between FBG levels and the risk of macrosomia. Furthermore, the employment of two-piecewise regression models facilitated the identification of a potential threshold for the FBG-macrosomia relationship. However, our study is constrained by its retrospective design, which relies on existing data and may be prone to bias and confounding variables. Additionally, our study population was confined to women who delivered at a single hospital, potentially limiting the generalizability of our findings. Moreover, gestation represents a multifactorial biological phenomenon wherein various determinants may substantially impact perinatal outcomes. While our analysis incorporated adjustments for key covariates including maternal age, educational attainment, and BMI, residual confounding may persist due to unaccounted variables such as pharmacotherapeutic interventions, habitual physical activity patterns, and nutritional exposures, which could potentially influence the observed associations.

In conclusion, our findings underscore the significance of glycemic control in preventing macrosomia. There is a nonlinear relationship between FBG levels during pregnancy and the risk of macrosomia in GDM women, with a potential threshold effect at 8.037 mmol/L. Below the threshold, macrosomia prevalence markedly rises with elevated FBG levels, whereas above it, the association loses significance, implying a potential saturation at very high glucose levels. Our study provides novel insights by identifying a distinct threshold for this risk, thereby contributing to a more nuanced understanding of the intricate relationship between FBG levels and fetal growth in GDM.

## Data Availability

The raw data supporting the conclusions of this article will be made available by the authors, without undue reservation.
